# DALY trend and predictive analysis of COPD in China and its provinces: Findings from the global burden of disease study

**DOI:** 10.3389/fpubh.2022.1046773

**Published:** 2022-12-23

**Authors:** Mimi Zhai, Qin Jiang, Sushun Liu, Jianhai Long, Dan Zhang, Chutong Ren, Yi Gong, Yamin Li

**Affiliations:** ^1^Clinical Nursing Teaching and Research Section, The Second Xiangya Hospital, Central South University, Changsha, Hunan, China; ^2^Xiangya Nursing School, Central South University, Changsha, Hunan, China; ^3^Department of General Surgery, The Second Xiangya Hospital, Central South University, Changsha, Hunan, China; ^4^Department of Respiratory, Beijing Tiantan Hospital, Capital Medicine University, Beijing, China

**Keywords:** COPD, GBD, DALY, China, provinces

## Abstract

**Background:**

Chronic obstructive pulmonary disease (COPD) is the most common chronic respiratory disease in the world, especially in China. Few studies have explored the trend of COPD in China and its provinces. This study aimed to demonstrate and predict the trend of COPD DALY in China and its provinces based on the global burden of disease (GBD) data.

**Methods:**

The data on COPD disability-adjusted life year (DALY) were collected from GBD 2017, GBD 2019, and the National Bureau of Statistics of China. The age-standardized rate (ASR) was used to evaluate the trend of COPD DALY by gender, age, and risk factors in China and its provinces. In addition, the trend of COPD considering the aging population in the next 10 years was also predicted.

**Results:**

In China, the COPD DALY was 20.4 million in 2017, which decreased to 24.16% from 1990 to 2017. Most provinces showed a downward trend, with the exception of Taiwan which increased by 127.78%. The ASR of DALY was 1445.53 per 100,000 people in 2017 and demonstrated a significant decrease. Among all provinces, only Taiwan (97.78%) and Hubei (2.21%) demonstrated an increased trend of ASR. In addition, Tibet ranked third with a decline of 56.95%, although its ASR was the highest in 1990. Smoking and air pollution were the main risk factors for COPD and varied with regions, gender, and age. The proportion of COPD DALY attributable to smoking was higher in the middle-aged and elderly male population and did not decrease in China. Moreover, the ASR attributable to air pollution of the elderly decreased significantly in China. Socio-demographic index (SDI) and educational level were also found to be related to ASR. By predicting the ASR trend in the next 10 years, we found that the ASR attributable to smoking might increase significantly among men. The ASR attributable to air pollution showed a significant decrease in women. Unfortunately, ASR attributable to second-hand smoke was found to increase in women.

**Conclusion:**

Chronic obstructive pulmonary disease is the leading contributor to the burden of global diseases. Although China and its provinces demonstrated a downward trend of COPD DALY, some provinces still faced challenges. Moreover, ASR attributable to risk factors was different in regions, gender, age, and years. The predicted trend of COPD was also different. Therefore, more targeted strategies should be formulated to reduce the burden of COPD in China and its provinces.

## Introduction

Chronic obstructive pulmonary disease (COPD) is the most common chronic respiratory disease in the world, with a high mortality rate (1). Previous studies have found that nearly 3 million people died from this disease, accounting for around 5.4% of all deaths worldwide, ranking fourth among all causes of death worldwide (2). About 90% of COPD deaths still occurred in low- and middle-income countries (2, 3). In addition, the morbidity and mortality of COPD in China are very high, ranking third among the causes of death in China (4, 5). As the aging population intensifies and lifestyle changes in China, the incidence and mortality of COPD will continue to increase and lead to more serious economic, social, and medical burdens (6). Although previous cross-sectional studies and systematic reviews have reported the epidemic trend of COPD in China, these studies have certain limitations, such as poor representativeness of sample size, heterogeneity of evaluation methods, and lack of some provinces' data (7, 8). In addition, patients over 40 years old were selected as the study population in most studies (7, 8). However, recent studies showed that COPD was becoming more and more common among young people (9). At present, rare studies explored the temporal trend of COPD in China and its provinces, especially the distribution and trend of risk factors of COPD in China.

The Global Burden of Disease (GBD) provides a comprehensive and consistent measurement framework for global health researchers and policymakers. In addition, it systematically quantified the health losses caused by 369 diseases and injuries and 87 risk factors in 204 countries and regions from 1990 to 2019 (10–12). As the GBD study and the National Bureau of Statistics of China improved the data and methods, it is now possible to study the trend and potential causes of COPD in time. In this study, we discussed the temporal trend of disability-adjusted life year (DALY) in China and its provinces. Moreover, we studied the attributable risk factors of COPD and predicted the trends of COPD. By incorporating these subnational data, we showed the COPD trend and risk factors in China. These results can be used to formulate actions to reduce the burden of COPD in China and its provinces.

## Methods

### Data source

The data from the GBD 2017 study and GBD 2019 study were used in our study. In the analysis at the provincial level, we used data from Zhou et al. study (5), GBD 2017, and the National Bureau of Statistics of China. When analyzing the trend, risk factors, and predicted trend of COPD, we used data from the GBD 2019 study. The data used in this study do not contain any information related to patients' privacy or any individually identifiable information. In addition, we followed the Creative Commons Attribution-Non-Commercial-NoDerivatives 4.0 International License and Section 7 of the University of Washington's Website Terms and Conditions of Use.

### Disability-adjusted life-years

Disability-adjusted life year (DALY) is a comprehensive indicator of health loss caused by lethal and non-lethal results. DALY is calculated as the sum of years lived with disability (YLDs) and years of life lost (YLLs). Losing a year's health is equivalent to a DALY. The gap between the current health status and the overall health status is reflected in the sum of all DALY in all populations.

### Age-standardized rate

Age-standardized rate (ASR) is an indicator to measure the changes in disease patterns in the population distribution. The ASR is very suitable for the comparison of different groups of people. Specific methods of calculating ASR are the same as that reported in previous studies (13–15).

### Socio-demographic index

The socio-demographic index (SDI) is an index that comprehensively assesses the development level of the total fertility rate, lagged distributed income per capita, and mean years of education over the age of 15 years in regions. Each part is scored from 0 to 1, and the SDI score is calculated as the geometric average of each part. Regions were grouped as low, low-middle, middle, high-middle, and high SDI regions according to SDI.

### Trend prediction

In our study, Hyndman Khandakar algorithm was used to calculate the parameters (p, d, q) of the ARIMA model by the “xtarimau” command of STATA 16.0. The best matching model was selected according to log-likelihood function (LLF), akaike information criterion (AIC), and schwarz information criterion (SIC). Using the selected model, the ASR of COPD DALY in the next 10 years was predicted. On the whole, the ARIMA (p, d, q) model was used in our study to predict the trend of COPD.

### Statistical analysis

The burden of COPD was estimated in different gender and all age groups by using GBD 2017 at the provincial level and using GBD 2019 at the national level. Our estimation studied the DALY and its trend in 34 province-level administrative units in China and associated risk factors at the national level. In order to explore the influencing factor of COPD DALY, a correlation analysis between SDI, education level, smoking, ambient particular matter pollution, and ASR in China was made. General methodologies for estimations have been published in our previous research (13–15). All data were analyzed by R software (Version 3.5.3, R Rore team). A *p*-value of <0.05 was considered to be statistically significant.

## Results

### DALY of COPD in China and its provinces

**Figure 1:**
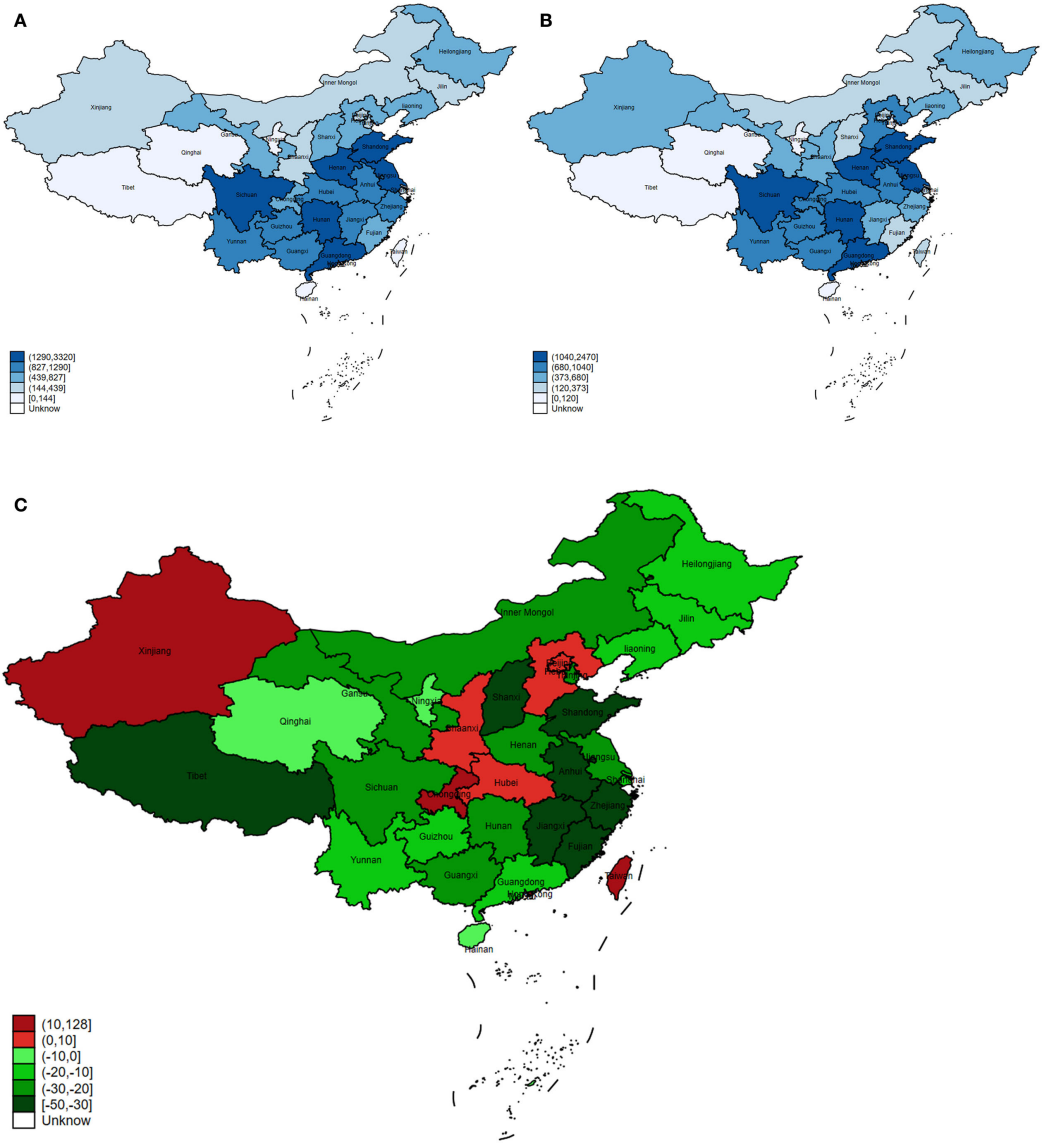
The trend of COPD DALY in China and its provinces. **(A)** The DALYs in China and its provinces in 1990. **(B)** The DALYs in China and its provinces in 2017. **(C)** The change of DALY cases in China and its provinces from 1990 to 2017.

In 2017, there were 20.4 million COPD DALYs at the national level in China (Figures 1A, B). The number of DALYs decreased by 24.16% from 1990 to 2017 in China, and it varied in different provinces of China (Figure 1C). The highest number of DALYs was found in Sichuan in 1990 and in 2017 (Figures 1A, B). On the contrary, the region with the lowest number was Hong Kong, but it showed an increasing trend with a growth rate of 18.25% from 1990 to 2017 (Figures 1B, C). During this period, the number of DALYs in most provinces demonstrated a downward trend, such as Zhejiang, Jiangxi, and Shandong (Figure 1C). Taiwan showed the largest increase in DALY with an increase of 127.78% from 1990 to 2017 (Figure 1C). Notably, for the three provinces (Sichuan, Shandong, and Hunan) with the largest number of DALYs, all their DALYs showed a significant decline trend, and Shandong province showed the most significant decline rate of 44.03% among them (Figures 1B, C). In addition, Zhejiang and Jiangxi provinces ranked the top two in China with a decrease of 49.06% and 47.29% in the number of DALYs, respectively (Figure 1C). The DALY in Ningxia, Hebei, Hubei, and Shaanxi provinces remained stable with a change of <5% (Figure 1C).

### ASR of COPD DALYs in China and its provinces

**Figure 2:**
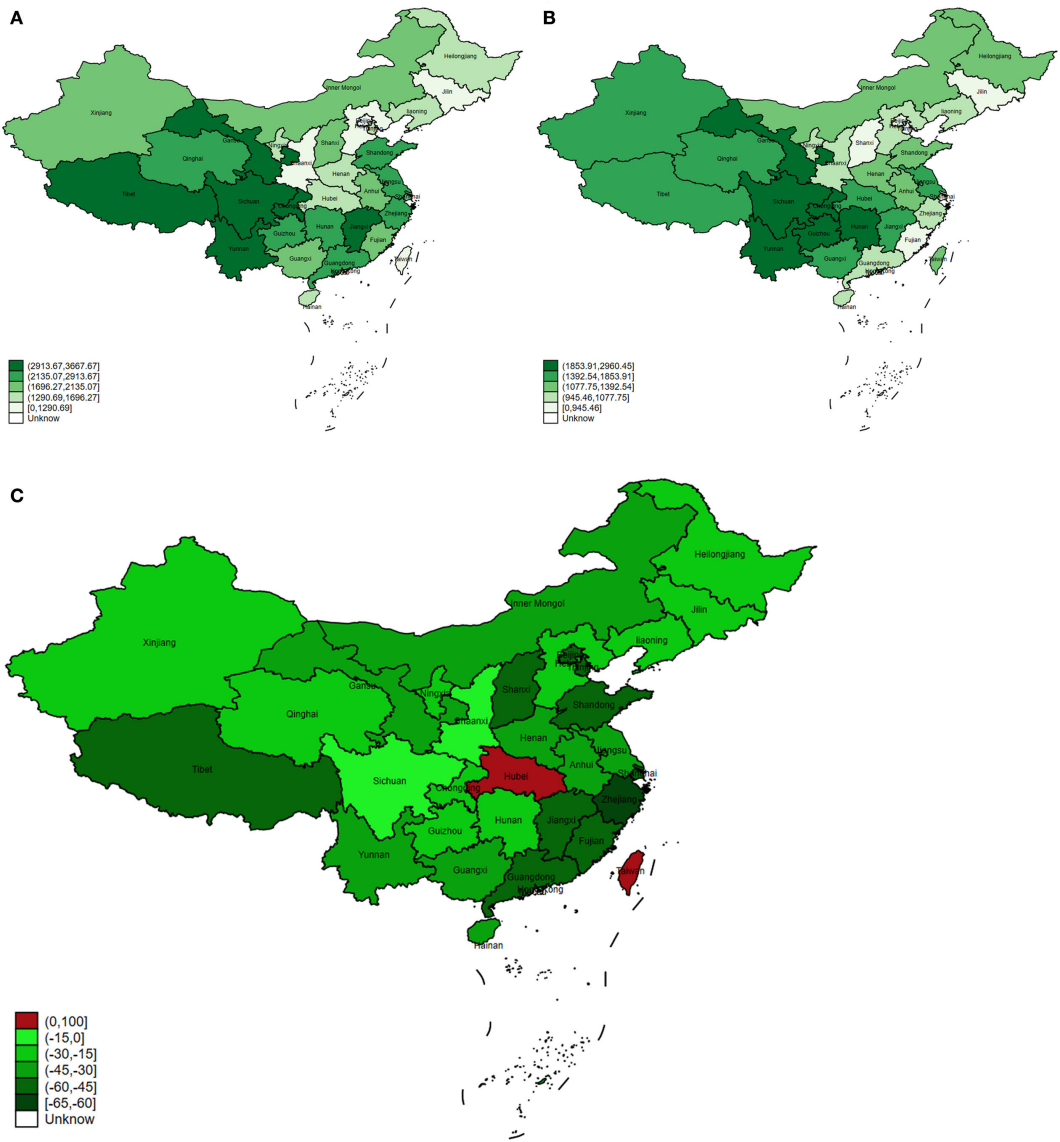
The trend of ASR of COPD DALY in China and its provinces. **(A)** The ASR of COPD DALY in China and its provinces in 1990. **(B)** The ASR of COPD DALY in China and its provinces in 2017. **(C)** The change of ASR in China and its provinces from 1990 to 2017.

The ASR of DALY dropped from 2250.82 per 100,000 people in 1990 to 1445.53 per 100,000 people in 2017 in China. A significant downward trend was observed in the ASR of DALY during this period. In 1990, Tibet had the highest ASR and its ASR showed third decline rate with a value of 56.95% (Figures 2A–C). In 2017, the ASR of Tibet ranked tenth among all provinces (Figure 2B). On the contrary, Chongqing had the second highest ASR in 1990 and 2017, although it decreased by 26.39% from 1990 to 2017 (Figures 2A–C). In addition, Taiwan had the lowest ASR in 1990, but due to the highest change in ASR (97.78%), the ASR increased sharply in 2017, ranking 14th among all provinces (Figures 2A–C). Among all provinces, only Taiwan (97.78%) and Hubei (2.21%) demonstrated an increased trend of ASR (Figure 2C). The most significant decrease trend of ASR was found in Zhejiang (63.44%), followed by Shanghai (58.00%) (Figure 2C).

**Figure 3:**
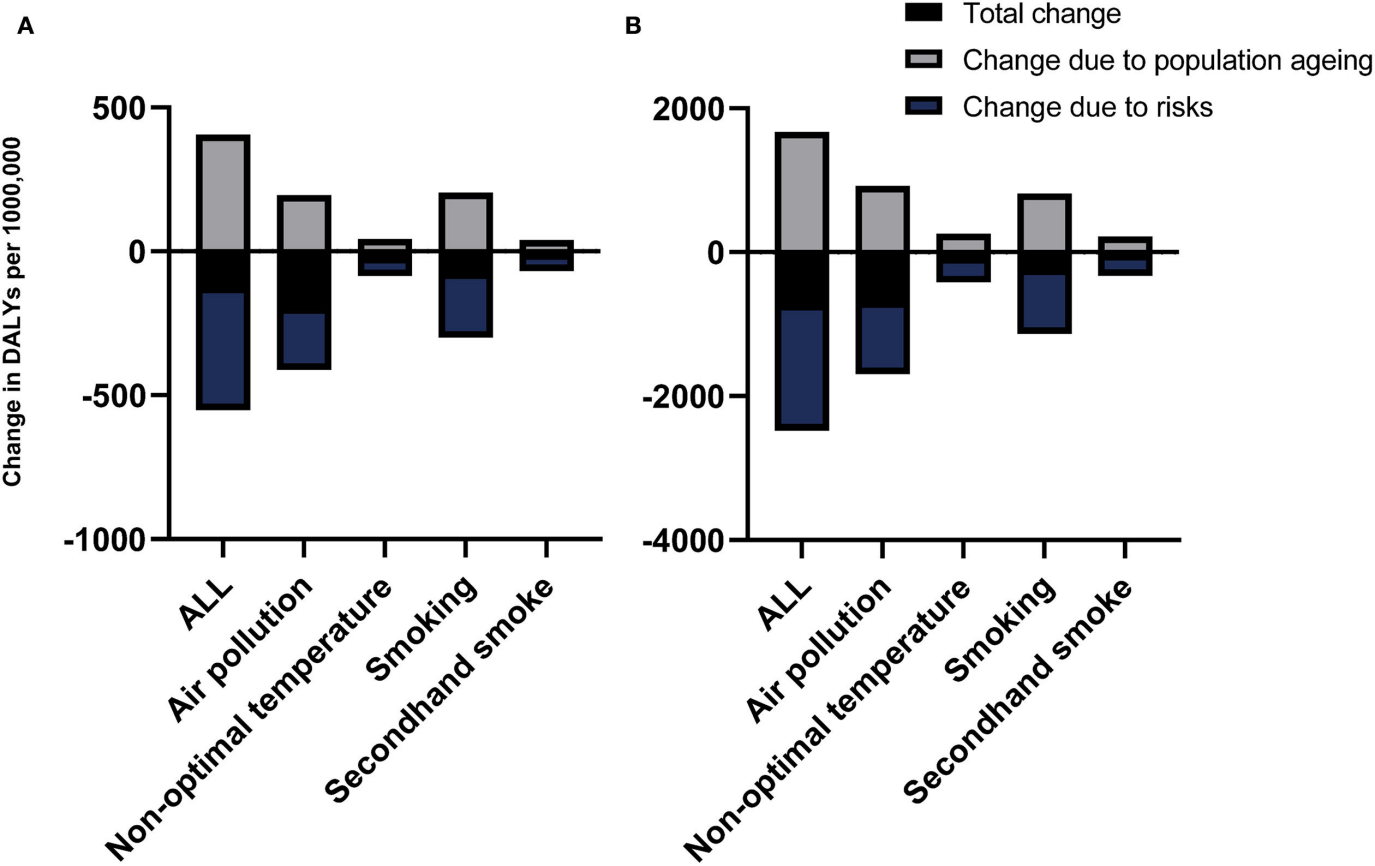
The change in ASR of COPD DALY considering the population aging in different risk factors in the world **(A)** and China **(B)**.

Furthermore, we studied the ASR of COPD DALY considering the aging population. Although the aging population had caused an increase in ASR, the overall ASR still showed a downward trend in China and in the world (Figures 3A, B).

### Influencing factors for ASR of COPD DALY

**Figure 4:**
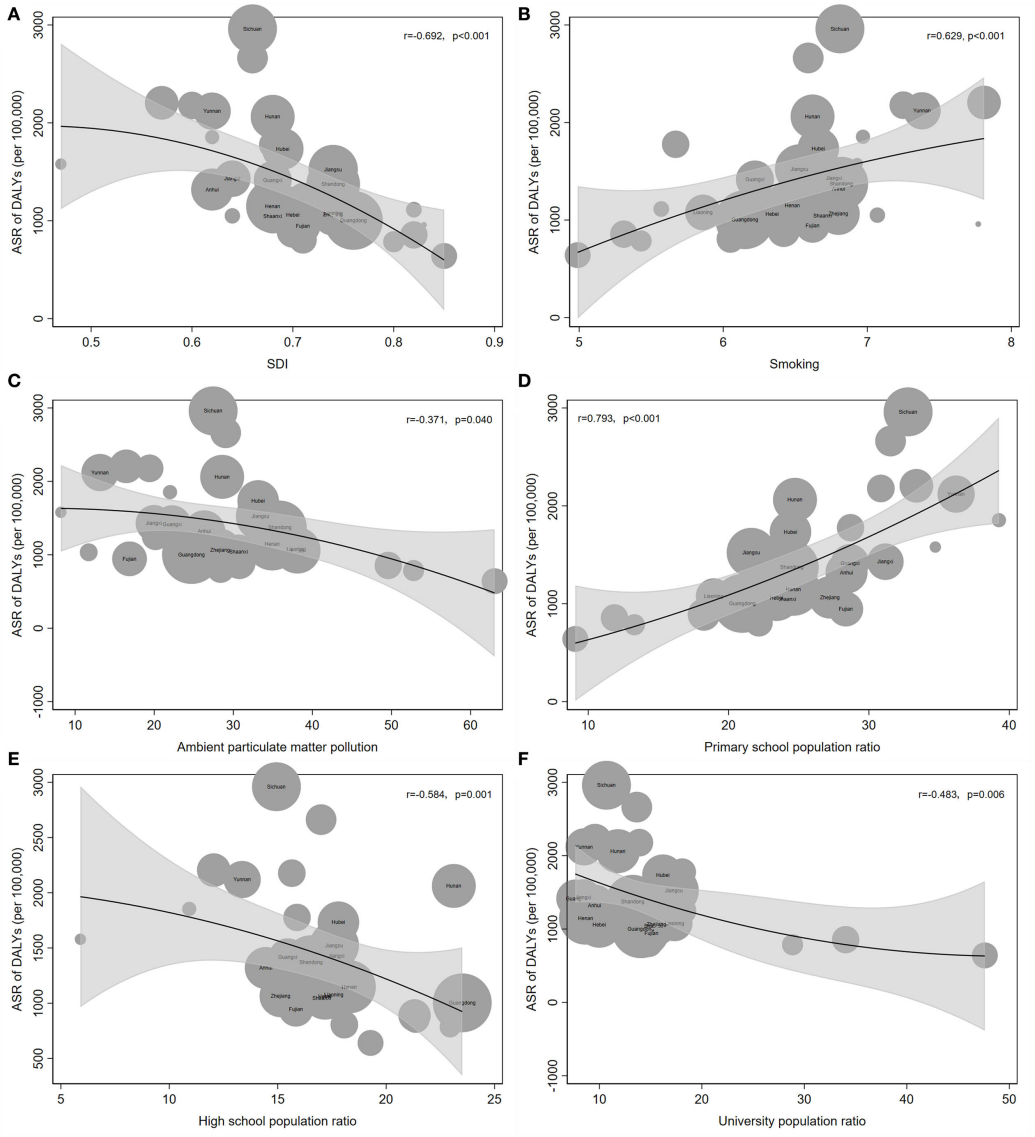
Influencing factors for ASR of COPD DALY. **(A)** SDI and ASR of COPD DALY were negatively correlated. **(B)** Smoking was positively related to ASR. **(C)** Ambient particulate matter pollution was negatively correlated with ASR. **(D)** Primary school population ratio was positively correlated with ASR. **(E, F)** High school population ratio **(E)** and university population ratio **(F)** were both negatively correlated with ASR.

In general, the SDI and ASR were negatively correlated in China. Among the low-middle SDI regions, the ASR of Gansu was significantly above average (Figure 4A). Among the middle SDI regions, the ASR of Sichuan, Chongqing, and Hunan was higher than the average, especially in Sichuan and Chongqing (Figure 4A). On the contrary, the ASR in Shanxi and Ningxia was lower than the average (Figure 4A). Different from the provinces with low-middle SDI, provinces with middle-high SDI mainly experienced below-average conditions, such as Jilin, Shanxi, and Fujian (Figure 4A). The ASR in provinces with high SDI was basically at the average level (Figure 4A).

With the increase in the smoking index, the ASR of COPD DALY in each province increased gradually (Figure 4B). Among all provinces, Sichuan and Chongqing had much higher ASR than the average according to the smoking index (Figure 4B). In addition, the ASR of Hunan, Xinjiang, and Gansu was slightly higher than the average level. On the contrary, the ASR of Macau, Ningxia, Zhejiang, Shaanxi, Shanxi, and Jilin was lower than the average (Figure 4B). Notably, Macau suffered from a high smoking index, but the ASR was significantly below average. However, Xinjiang showed the opposite result (Figure 4B).

Notably, ambient particular matter pollution was negatively correlated with ASR of COPD DALY. Sichuan and Chongqing had a significantly higher ASR than the average level (Figure 4C). On the contrary, the ASR of Fujian, Jilin, and Shanxi were significantly lower than the average level (Figure 4C).

Finally, the relationship between education level and ASR of COPD DALY was analyzed. With the increase in the primary school enrollment rate, the ASR of DALY was also rising (Figure 4D). However, the ASR decreased, when the attendance rate of high schools and universities increased (Figures 4E, F).

### Risk factors for COPD

**Figure 5:**
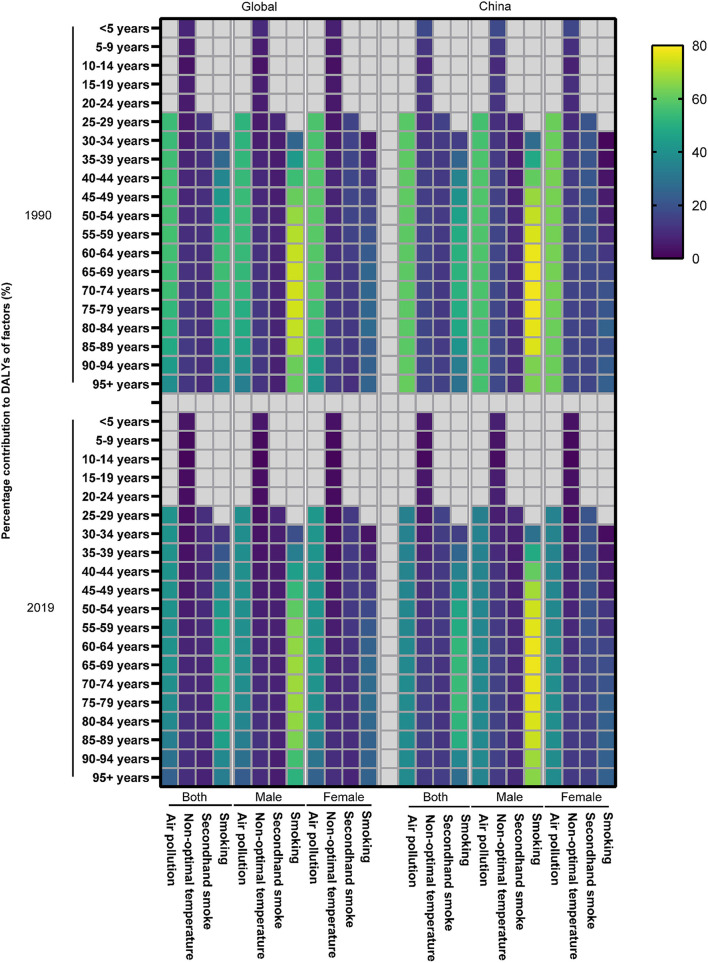
The contribution of risk factors to DALY of COPD in 1990 and 2019.

The DALY of COPD attributed to risk factors in total, men and women were studied from 1990 to 2019. Air pollution and smoking were the main risk factors for COPD and varied with age (Figure 5). In the middle-aged and elderly male population, smoking was the most important risk factor among all the risk factors (Figure 5). In addition, the proportion of smoking decreased around the world, but not in China (Figure 5).

**Figure 6:**
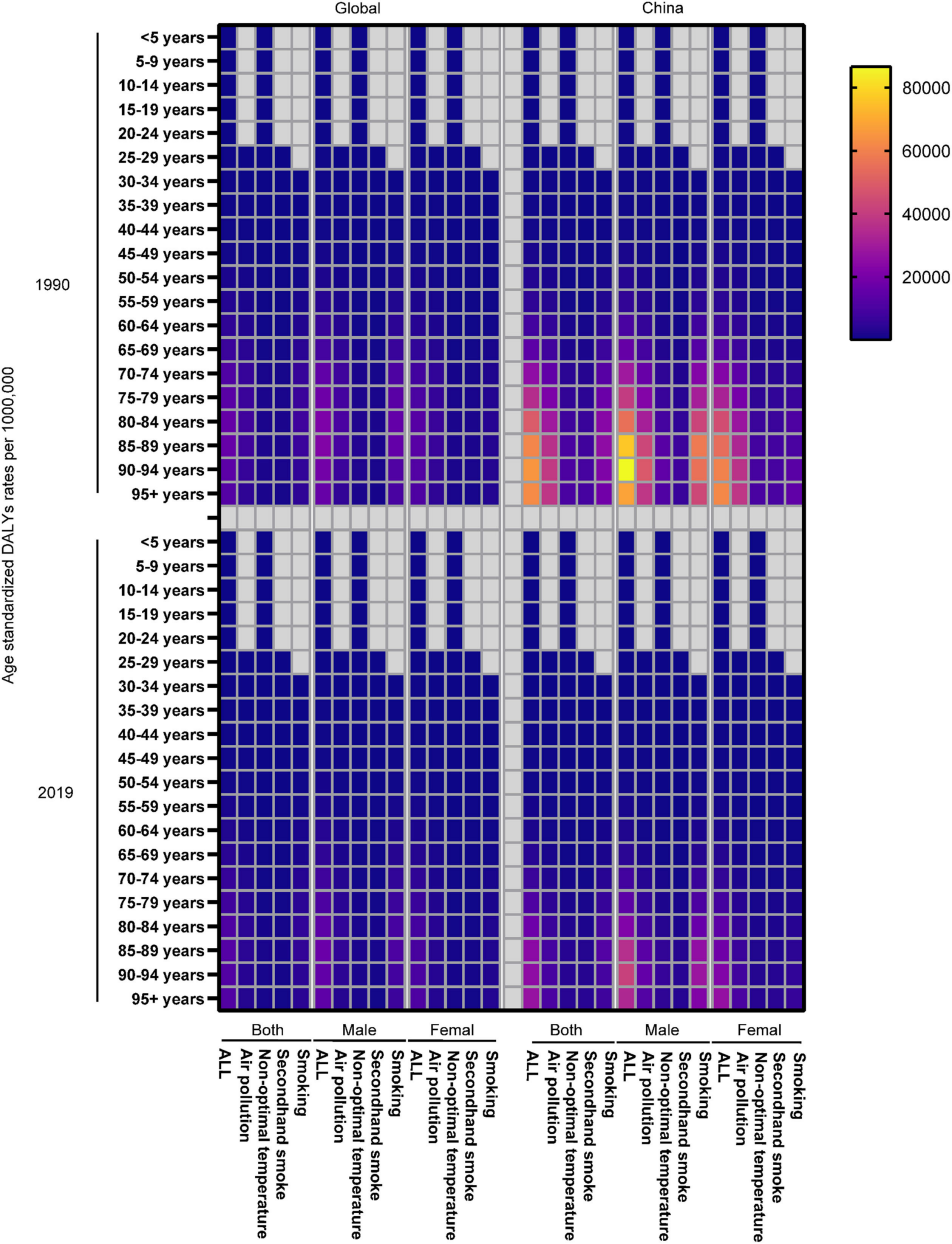
The ASR of COPD DALY attributable to risk factors in different ages in 1990 and 2019.

**Figure 7:**
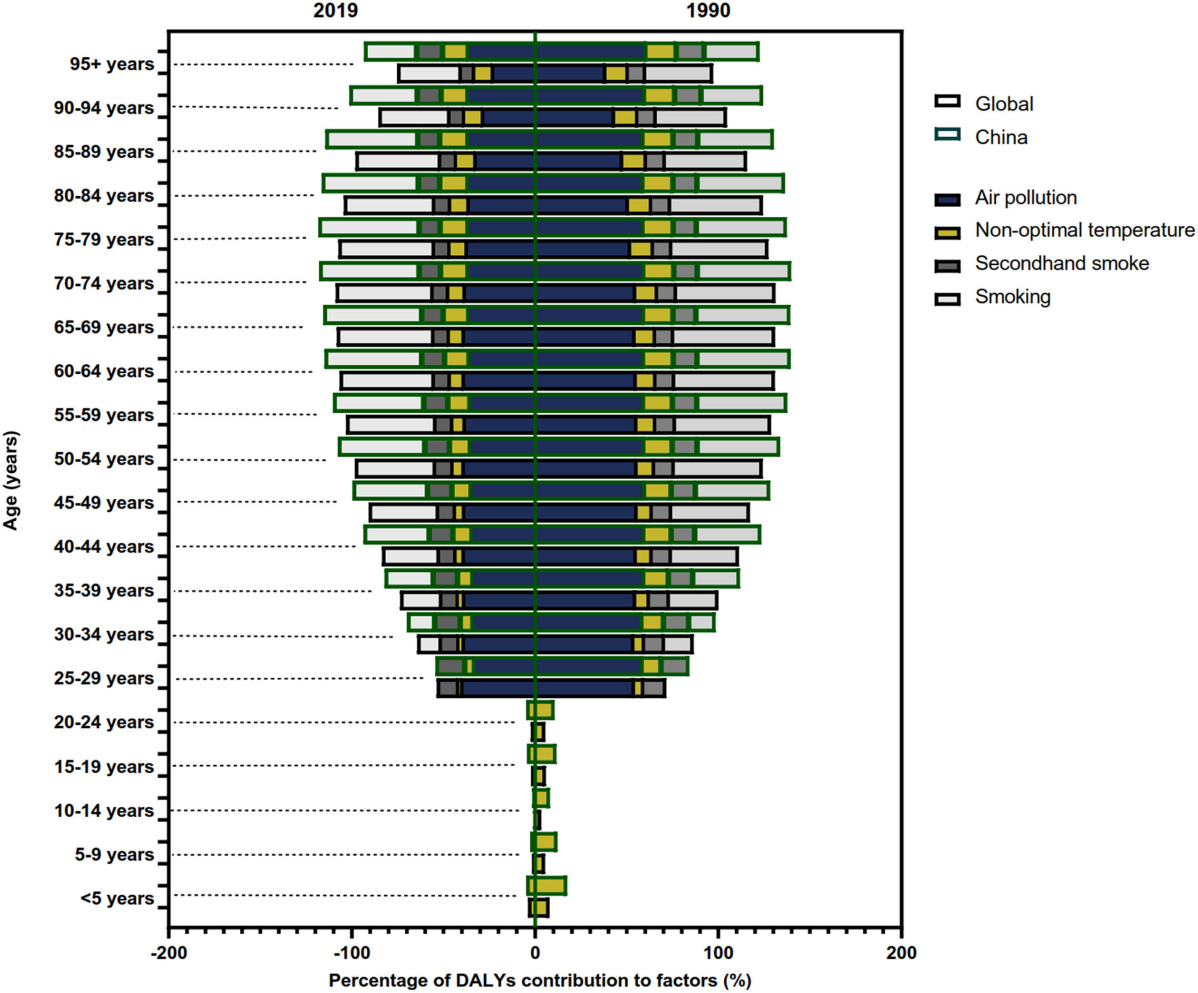
The contribution of risk factors to DALY of COPD in China and the world in different ages.

Similarly, the ASR of COPD DALY attributable to air pollution and smoking demonstrated a significant downward trend both in China and around the world compared with other risk factors, although the aging population led to an increase in ASR (Figures 3A, B). In addition, the ASR of COPD DALY attributable to all risk factors and air pollution in China decreased significantly among the elderly (Figure 6). The air pollution's contribution to DALY remained stable among men and women in different ages (Figure 7, Supplementary Figures S1, S2). However, with the increase in age, smoking's contribution to DALY first increased and then decreased (Figure 7). Besides, smoking's contribution to DALY was higher among men in China and around the world (Supplementary Figures S1, S2). In women, the percentage was also lower in China compared with the world (Supplementary Figures S1, S2).

### Prediction for the ASR of DALY trend of COPD

**Figure 8:**
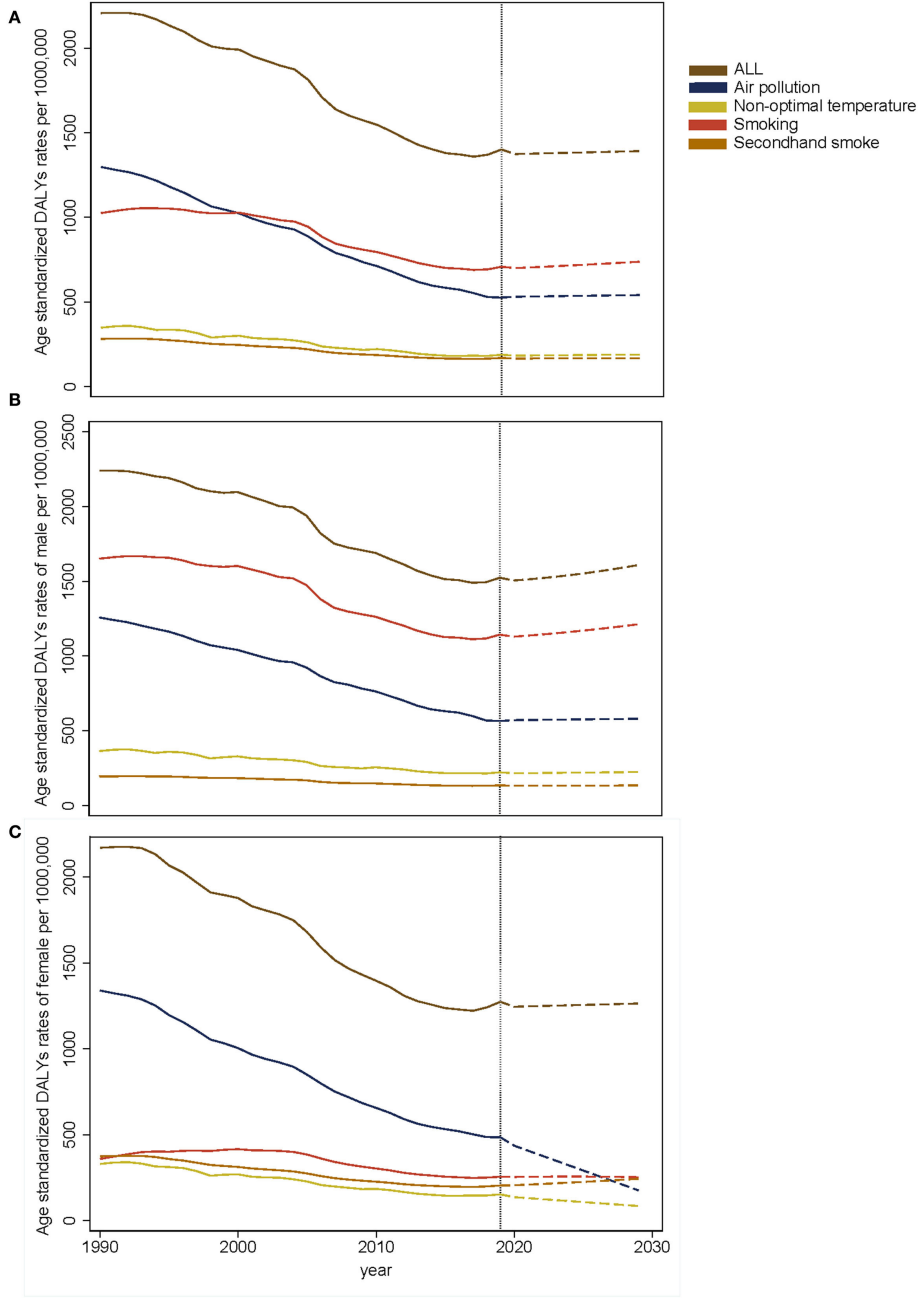
The prediction of ASR of COPD DALY in all **(A)**, male **(B)**, and female **(C)**.

The trends in observed and predicted ASR of COPD DALY are shown in Figure 8. In China, the ASR decreased, but we predicted that it might remain stable in the next 10 years (Figure 8A). In addition, the ASR of DALY might slightly increase in men, while it remains stable in women in the future (Figures 8B, C). The ASR attributable to smoking might increase slightly in the next 10 years, with a relatively large increase for men and a stable increase for women (Figures 8B, C). The ASR attributable to air pollution showed a minor increase in all and in men but decreased significantly in women in the next 10 years (Figure 8). Similarly, the ASR attributable to non-optimal temperature decreases in women in the next years, although it remained stable in men and in all people (Figure 8). Unfortunately, the ASR attributable to second-hand smoke was found to increase in women in the next 10 years (Figure 8C).

## Discussion

COPD is the most common chronic respiratory disease and the leading contributor to the global burden of diseases. The DALY of COPD ranked from eleventh to sixth during this period among all causes (1, 16). China has been facing the challenge of COPD for a long time, especially in the central provinces of China. In addition, the COPD DALY attributable to risk factors varied in gender, age, and regions. Thus, in order to establish more targeted preventions and control policies, we first revealed the pattern and trend of COPD burden at provincial and national levels. Moreover, we revealed the risk factors of DALY COPD by sex and age. Finally, we predicted the trend of COPD in the next 10 years. By comparing the DALY and its trend in different provinces, we can evaluate the effects of the current policies. By analyzing the COPD attributable risk factors, we can provide the basis for policymaking.

Chronic respiratory disease accounted for 4.7% of DALYs in the world, of which COPD accounted for two-thirds of the total (17). From 1990 to 2017, the number of COPD DALY decreased by 24.16% in China, with a downward trend in most provinces. The decline might be related to a series of factors, including effective anti-smoking measures, improvement of environmental pollution, promotion of clean energy and working environment, and improvement of COPD treatment. Moreover, China has improved its citizens' medical services. Recent healthcare reform has expanded the population covered by health insurance and reduced healthcare costs. The new rural cooperation insurance enabled patients with COPD, especially patients in rural areas, to be diagnosed and treated in the early stage (18). In addition, China has also promulgated the “Healthy China Action Task (2019–2030),” half of which are related to the prevention and treatment of respiratory diseases (19). However, the aging problem in China is becoming more and more serious, and the risk of COPD among the elderly is much higher than that of other people (4, 20). This poses new challenges for the prevention and treatment of COPD. In our study, an increase of more than 20% in the number of DALYs was found in Taiwan, Xinjiang, and Chongqing. It might be related to factors such as population growth and aging, high prevalence of smoking, biomass fuel use, increased air pollution, and improvement of diagnosis. Notably, the number of DALYs in Shandong province ranked second in 1990 and third in 2017. However, it dropped by 44.03% during this period, ranking third among all provinces. The high DALY in Shandong might be related to population, smoking, low medical level, and air pollution. With the development of the economy, improvement of medical level, and living habits, the DALYs of Shandong demonstrated a great decline. Moreover, the basic public health services and the new rural cooperative medical system also contributed to the decline of DALYs in Shandong (21). Unlike Shandong provinces, Zhejiang's DALY had the highest decline in China. This might be related to stricter anti-smoking measures, higher educational levels, easier access to medical services, a more developed economy, and a deeper understanding of COPD (22).

In order to avoid the impacts of provinces' population on DALYs, we further used the ASR of DALY to study the trend of COPD in provinces. By adjusting the difference in age and population, ASR can be used for comparison in different provinces and different periods. In 2017, Beijing showed the lowest ASR of DALY. This might be related to stricter smoke control measures, higher medical levels, lower smoking rates, and better medical insurance (23). On the contrary, Sichuan and Tibet, respectively, showed the highest ASR in 2017 and 1990. In Tibet, this might be caused by the use of biofuels, low weight due to malnutrition, and poor medical conditions (24). In Sichuan, this might be related to the high smoking rate, living habits, educational levels, and economic levels (25, 26). Moreover, the aging population in Sichuan also might affect the ASR. With the development of the economy, medical care, and the use of clean energy in Tibet, the ASR showed an obvious downward trend, although it was highest in 1990. Similar to the change in Zhejiang's DALY, it also showed the highest decrease in ASR of COPD DALY. The significant decrease in Zhejiang was attributed to economic and medical development and policy improvement.

Furthermore, we also studied the influencing factors and risk factors for ASR of COPD DALY. Previous studies found that there was a significant negative correlation between SDI and ASR of mortality and DALY worldwide (27, 28). Similarly, a negative correlation between SDI and ASR of COPD DALY was found in our study. It pointed out that provinces with high SDI obtained better access to health services and treatments. In addition, high SDI provinces implemented various strategies to reduce air pollution, which helped to reduce the ASR of COPD DALY.

Smoking has been identified as the most important risk factor for COPD. The epithelial cell, extracellular matrix, and blood supply in the lung might be destroyed by exposure to smoke (29, 30). In our study, the ASR of DALY might increase slightly in the next 10 years, especially among men, although it declined from 1990 to 2019. The increasing ASR of COPD DALY attributable to smoking might be associated with high smoking rates among young people, particularly among higher vocational students. The data obtained from the Chinese Center for Disease Control and Prevention (CDC) demonstrated that the smoking rate of higher vocational students was as high as 21.2% in men and 11.6% in all in 2021. Moreover, the smoking rate among people over the age of 15 remained stable. With the population growth, the number of smokers increased by 15 million from 5 years ago to 316 million. Moreover, the data obtained from CDC demonstrated that the number of cigarettes increased by one every day compared with 5 years ago. In addition, the increase in the use of electronic cigarettes was also the reason for the increased ASR. Apart from smoking, second-hand smoke was also an important contributing factor. Our study demonstrated that ASR attributable to second-hand smoke might increase in women in the next 10 years. Studies also found that more than one-third of children and non-smokers were exposed to second-hand smoke (27, 31). Thus, more strategies such as economic, cultural, media-based, and family functioning measures should be used to control smoking (32). Moreover, because of the lag effect of COPD intervention, relevant strategies should be implemented earlier.

Air pollution is another risk factor for COPD. In China, the considerable increase in emission has become a major social problem (33). With rapid economic development and the acceleration of urbanization in recent years, our government has taken effective measures to reduce air pollution (34). Interestingly, a study found that ambient particulate matter pollution related ASR of COPD DALY reached the highest level when SDI was 0.5 and began to fall when SDI exceeded 0.5 (28). Our study also found that there was a weak negative correlation between ambient particulate matter pollution and ASR of DALY. There are several reasons. First, with the increase in pollution, more attention has been paid to reducing the ASR of COPD DALY. Second, air pollution includes ambient particulate matter pollution, house air pollution, and ambient ozone pollution. The ambient particulate matter pollution's contribution to ASR might not be important. Moreover, regional differences, population structures, and lifestyles may also be the reasons for the decline in ASR of COPD DALY. Then, we also analyzed the reasons from the aspect of statistics. First, there is collinearity among many factors. For example, the region with a better economy might have more serious pollution, a lower smoking index, and a higher education level. Second, by multiple stepwise regressions, only the primary school ratio, not the air pollution was related to ASR. Finally, a previous study indicated that air pollution was correlated with SDI. However, air pollution was not related to ASR of COPD DALY, when SDI and pollution were included for analysis in our study. Our study demonstrated that the ASR of COPD DALY attributable to air pollution decreased in men and women. In addition, ASR is predicted to decrease in the next 10 years in women, although women are more sensitive to air pollution (32, 35). On the contrary, the ASR of COPD DALY attributable to air pollution in men increased slightly. Thus, various kinds of eco-friendly strategies should be implemented to reduce air pollution.

The educational level was also a risk factor for COPD (36). Further studies also found that people with a lower education level suffered from a higher risk of death due to exposure to PM2.5 (37). Similarly, our study found that the proportion of the high school and university population was negatively correlated with ASR of COPD DALY. Interestingly, a positive correlation was found between the primary school population ratio and ASR of COPD DALY. This might be related to the migration of these people to cities with more serious air pollution and higher smoking rates. Moreover, people with lower educational levels were more stressed in life and might smoke more cigarettes. Thus, improving the education level of the population and health education on COPD was of great significance for reducing the ASR of COPD.

## Conclusion

The COPD DALY and ASR of COPD DALY decreased in China and in most provinces, but some provinces still showed an increasing trend. In addition, the ASR attributable to each risk factor varied in regions, gender, age groups, and years. Our study helps to demonstrate the trend of COPD and the change in its attributable risk factors in China and its provinces. Furthermore, the predicted trend of COPD and its attributable risk factors were helpful for further understanding of COPD burden. Therefore, according to our study results, more targeted and effective measures could be formulated.

## Limitations

There are some limitations in our research. First, considering the difficulty of distinguishing DALY from COPD and DALY due to its comorbidities, DALY of COPD might be underestimated. Second, some patients with COPD were not included in the GBD database in some provinces in China, especially in underdeveloped areas. Additionally, based on the GBD database, it was impossible to study the effect of multiple risk factors on COPD. Therefore, more studies were needed to improve the result of COPD in China and its provinces, although the systematic and comprehensive methodological framework adopted by GBD had its advantages.

## Data availability statement

The datasets presented in this study can be found in online repositories. The names of the repository/repositories and accession number(s) can be found below: http://www.healthdata.org/gbd.

## Ethics statement

Ethical review and approval was not required for the study on human participants in accordance with the local legislation and institutional requirements. Written informed consent from the participants was not required to participate in this study in accordance with the national legislation and the institutional requirements.

## Author contributions

Study design: MZ, YG, and YL. Data collection: MZ, SL, and JL. Data analysis: JL, DZ, and CR. Figures: SL and QJ. Manuscript writing: MZ, YG, and SL. Manuscript proofing: MZ and YL. All authors contributed to the article and approved the submitted version.
