# Supplementary figures and images for: DALY trend and predictive analysis of COPD in China and its provinces: Findings from the global burden of disease study
Source: Front Public Health. 2022 Dec 23;10:1046773. doi: 10.3389/fpubh.2022.1046773 (PMC9816410; doi:10.3389/fpubh.2022.1046773)

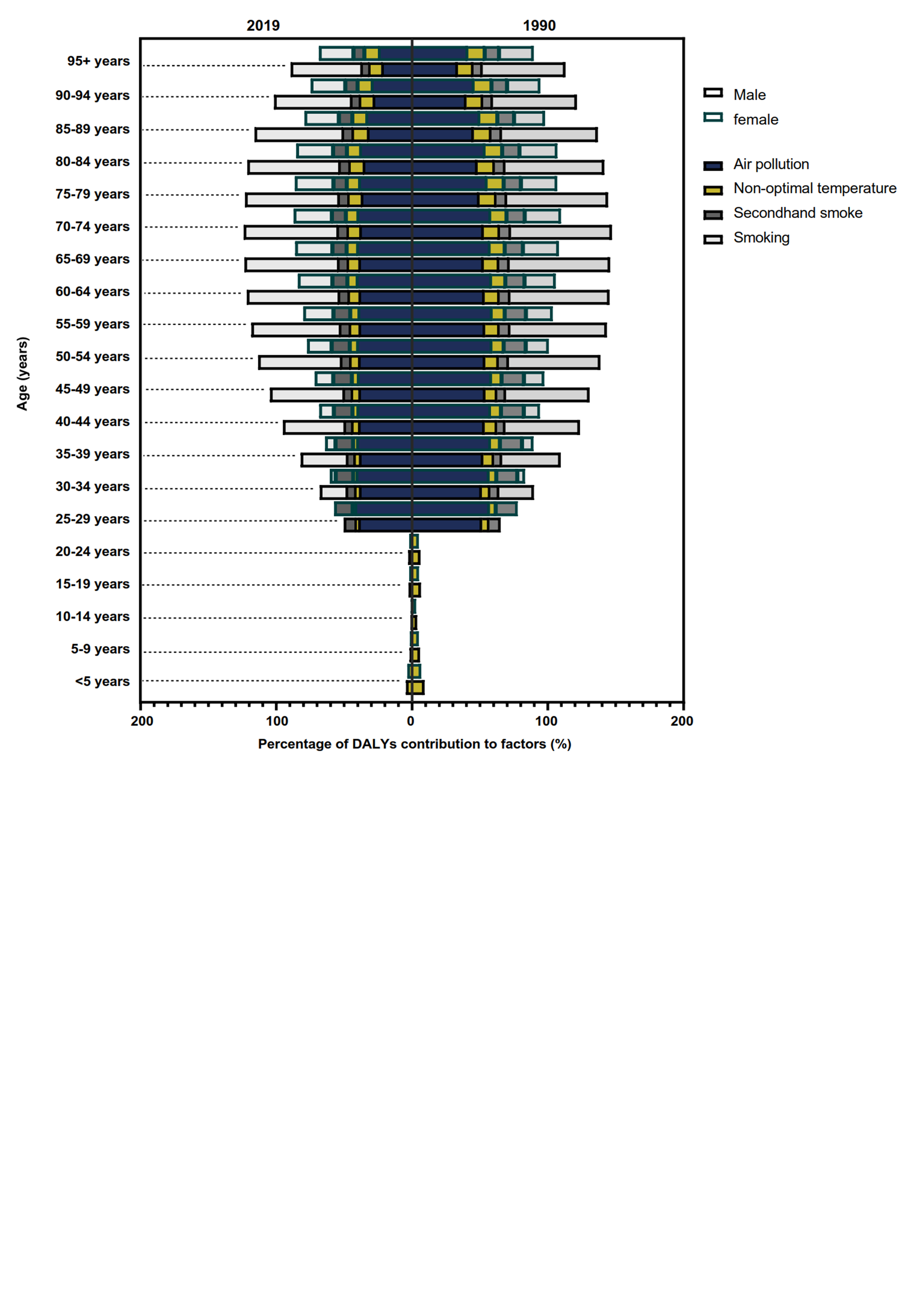

Supplement: Supplementary file 1 [file Image_1.tif]

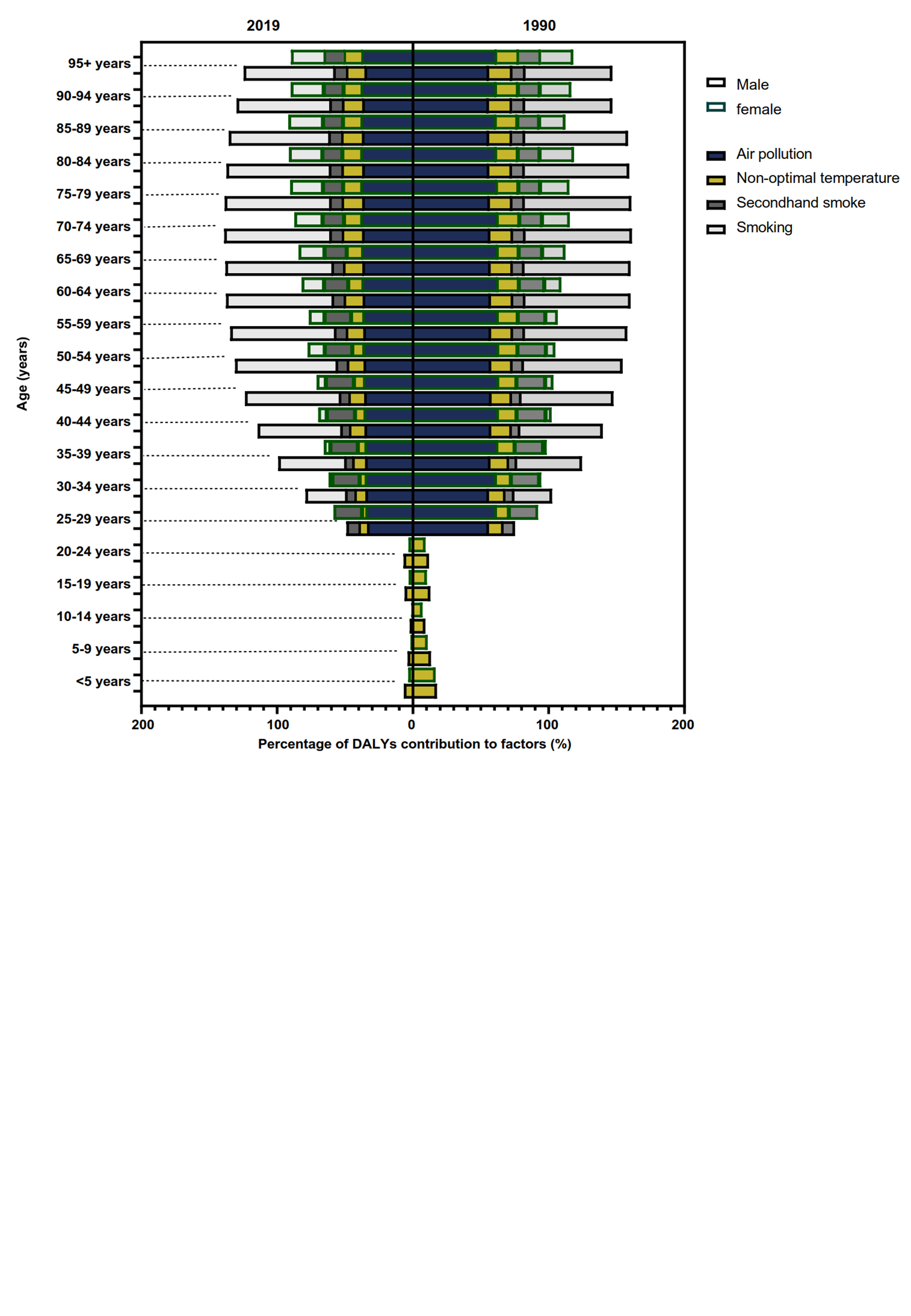

Supplement: Supplementary file 2 [file Image_2.tif]
